# Depression as a predictor of work resumption following myocardial infarction (MI): a review of recent research evidence

**DOI:** 10.1186/1477-7525-8-95

**Published:** 2010-09-06

**Authors:** Adrienne O'Neil, Kristy Sanderson, Brian Oldenburg

**Affiliations:** 1 School of Public Health and Preventive Medicine, Monash University, 89 Commercial Road, Melbourne, Victoria 3004, Australia; 2 Menzies Research Institute, University of Tasmania, Private Bag 23, 52 Bathurst St, Hobart, Tasmania 7001, Australia

## Abstract

**Background:**

Depression often coexists with myocardial infarction (MI) and has been found to impede recovery through reduced functioning in key areas of life such as work. In an era of improved survival rates and extended working lives, we review whether depression remains a predictor of poorer work outcomes following MI by systematically reviewing literature from the past 15 years.

**Methods:**

Articles were identified using medical, health, occupational and social science databases, including PubMed, OVID, Medline, Proquest, CINAHL plus, CCOHS, SCOPUS, Web of Knowledge, and the following pre-determined criteria were applied: (i) collection of depression measures (as distinct from 'psychological distress') and work status at baseline, (ii) examination and statistical analysis of *predictors *of work outcomes, (iii) inclusion of cohorts with patients exhibiting symptoms consistent with Acute Coronary Syndrome (ACS), (iv) follow-up of work-specific and depression specific outcomes at minimum 6 months, (v) published in English over the past 15 years. Results from included articles were then evaluated for quality and analysed by comparing effect size.

**Results:**

Of the 12 articles meeting criteria, depression significantly predicted reduced likelihood of return to work (RTW) in the majority of studies (n = 7). Further, there was a trend suggesting that increased depression severity was associated with poorer RTW outcomes 6 to 12 months after a cardiac event. Other common significant predictors of RTW were age and patient perceptions of their illness and work performance.

**Conclusion:**

Depression is a predictor of work resumption post-MI. As work is a major component of Quality of Life (QOL), this finding has clinical, social, public health and economic implications in the modern era. Targeted depression interventions could facilitate RTW post-MI.

## Introduction

### Relationship between myocardial infarction, depression and work

Depression is a common and debilitating condition which is often experienced after a heart attack [myocardial infarction (MI)]. It is estimated that approximately 15% of individuals will suffer major depression post-MI, with another 15-20% exhibiting mild to moderate symptoms [1]. Although depression may be transitory, there is evidence to suggest it can precede a cardiac event. For example, more than half of MI patients experience feelings of fatigue and general malaise in the months before infarction [2]. Despite its prevalence, depression often remains unrecognised and undiagnosed in this population. This may be due to issues such as brief hospitalisation periods (the average length of stay for MI is now 3-5 days [3]) and the fact that symptoms of depression and MI can overlap. Left untreated, co-morbid depression has a significant impact on recovery and functioning and is associated with increased morbidity and mortality, poorer clinical, behavioural and psychological outcomes, and reduced overall quality of life (QOL) [4].

Work is a major constituent of QOL. It plays an important role in the recovery and adjustment of patients post-MI, through its related constructs such as satisfaction, social value and productivity. With evidence to suggest survival rates are increasing, indeed many patients will resume work after experiencing a cardiac event; it is currently estimated that 80% of MI patients will return to work (RTW) post infarct within a 12 month period [5]. However, patients with cardiac depression are slower and less likely to RTW [6] than those without. For patients who have not resumed work by 12 weeks, the likelihood of doing so decreases by half [7]. Depression symptoms- both cognitive and somatic- can inhibit desire to resume employment, resulting in longer absences from the workplace. In patients who RTW, the benefits remain well documented; increased positive affect and fewer cognitive complaints [8]. However, those experiencing co-morbid depression are more likely to report poorer vocational functioning, social problems, increased absenteeism, presenteeism or early retirement. Despite this evidence, research investigating depression as a prognostic indicator of RTW post MI has produced inconsistent results in recent years [9].

### Existing evidence for depression as a predictor of RTW after MI

During the 1970 s and 80 s, RTW was considered a key indicator of the effectiveness of cardiac rehabilitation and patient recovery. Age, education, socio-economic status, severity of MI, and physical functioning were all implicated as strong moderators of RTW after a cardiac event. The latter was often used as a means by which to measure one's capacity and readiness to RTW (e.g. Dennis, 1988 [10]). However, during this time, the prognostic role of depression and psychosocial factors became of interest. Two key studies of this time [Hlatky et al (1986) and MÆland et al (1987)] found that depression recorded in hospitalised cardiac patients predicted poorer RTW outcomes, increased work disability and greater loss of employment [11,12]. Patients with co-morbid depression were also found to experience greater difficulties in occupational adjustment and deficits in other outcomes. MÆland et al (1987) further observed a linear relationship between RTW and levels of depression, concluding that increased depression severity was linked to poorer rates of RTW in MI patients [11].

More recently, although evidence has emerged that depression is a predictor of employment status up to a year after admission for patients with other cardiovascular (CVD) conditions, such as stroke [13], in MI populations it "cannot be assumed that factors identified over 25 years ago as predictors of return to work will be relevant in the modern era"[14]. There are several reasons for this. Longitudinal trends have indicated that survival rates after MI are increasing [15,16]. For example, data from the Atherosclerosis Risk in Communities (ARIC) study [1987 to 1994] indicated a decline in MI severity in the US [17]. This trend was further demonstrated for the period 1994-2002 [16]. Second, advances in procedures for diagnosis and treatment, i.e. imaging stress tests, Percutaneous Coronary Intervention (PCI) and stents, overall rates of revascularization (substantially increasing since 1993 [18]), and increased medication prescription [aspirin, Angiotensin-converting enzyme (ACE) inhibitors] [19] have led to changes in the management of cardiac patients. Third, trials investigating the role of depression post MI [20] have more likely been expressed using clinical and psychological markers over employment outcomes. Fourth, increased awareness about the prevalence of depression in this population has led to further research in this area in recent years. In light of the contemporary management of cardiac patients, and the subsequent implications on rates of discharge and RTW, recent studies need to be drawn on to determine if depression remains a predictor of work outcomes post MI.

The identification of depression as a predictor of work outcomes in MI patients is important. From a clinical perspective, facilitating RTW after MI may significantly reduce emotional distress [21]. From a societal perspective, shifts in social trends including increased life expectancy and financial instability, translating to longer working lives, require that barriers to workforce participation be identified. From a public health perspective, the increasing burden of coronary heart disease on western society, its augmented risk with age, and increased survival rates (e.g. up to 20 million people survive a heart attack globally each year [22]), highlight a need to implicate factors which facilitate workforce participation. From an economic perspective, depression as a sole condition accounts for 13.8 million work days lost in the UK [23] and 225 million days lost in the US, annually [24]. When co-existing with a chronic disease, depression can have even greater economic implications on the workforce.

The aim of our study was to determine whether depression remains a predictor of poorer work outcomes following MI by conducting a review of studies conducted in the past 15 years.

## Methods

### Search Strategy

The literature search aimed to identify articles which assessed work resumption as an outcome measure and depression as a primary prognostic variable in cardiac patients. Studies were identified using databases for medical, health, occupational and social sciences, with the intention to cover concepts identified by the authors in Table 1. Databases included PubMed, OVID, Medline, Proquest, CINAHL plus, CCOHS, SCOPUS, Web of Knowledge. Reference lists of relevant studies and reviews (identified using databases such as EBM Reviews, Cochrane DSR, ACP Journal Club, DARE, CCTR, CMR, HTA, and NHSEED) were also examined. Grey literature and web pages were examined using search engines such as Google Scholar. Previous recommendations for effective strategies in identifying prognostic studies [25] were also employed.

**Table 1:** Search concepts and terms

Concepts	Terms
Predictors	Determinants, factors, influences, risk, psychological, clinical, social, psycho social

Work resumption	Return to work, loss of work, absenteeism

Recovery	Cardiac rehabilitation, adjustment, lifestyle

Employment	Work, full time, part time, workplace, vocation, job content, work limitations, productivity, work outcomes

Quality of Life	Impairment, functionality, activity

Demographic information	Age, gender, education, socio economic status, income

Chronic disease	Myocardial Infarction, Acute Coronary Syndrome, Cardiovascular disease, Coronary Heart Disease, Coronary Artery Disease, depression, psychological distress, morbidity, co-morbidity

### Selection of studies

Articles were identified using this search strategy and reviewed for relevance by the first author and an independent reviewer (CR) between March and July, 2009. Abstracts were obtained for articles which potentially included: (i) application of depression measures (as distinct from 'psychological distress') and work status at baseline, (ii) examination and statistical analysis of *predictors *of work outcomes, (iii) cohorts with patients exhibiting symptoms consistent with ACS, (iv) follow up of work-specific and depression specific outcomes at minimum 6 months, (v) those published in English over the past 15 years. Full text articles were obtained for those appearing to meet criteria, where the following information was extracted from each: author, population, design, depression measure, definition of RTW, major findings, effect of depression as a predictor on RTW, other significant predictors of RTW post MI. Data were analysed through synthesis and quality assessment of this information, as the inconsistencies between study definitions of RTW and variety of instruments used to assess depression precluded formal meta-analysis. Using a framework for assessing internal validity used in other prognostic reviews [26], these articles were subject to application of a quality criteria (Additional file 1). Articles were systematically scored in reference to quality, to determine level of evidence. A score of 12 or more was considered high quality, 10-11 was considered moderate quality and nine or less was deemed low quality. The quality of articles was considered not as exclusion criteria but in the analysis of results.

## Results

**Figure 1:**
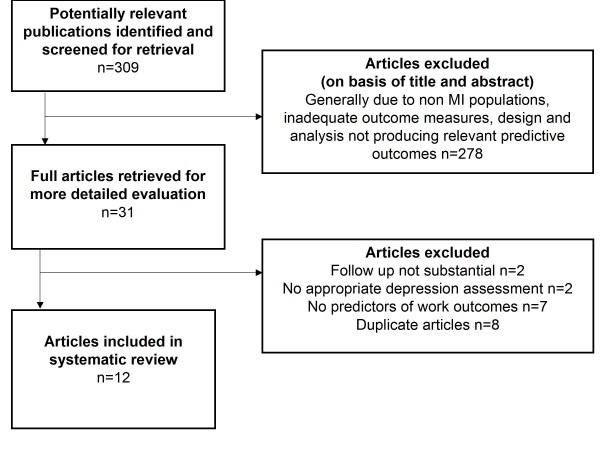
**Flowchart of search strategy results**.

Initial searches were conducted independently by AO and CR, yielding 1231 results; 309 of these articles were considered for inclusion from an initial review, and their abstracts obtained. After screening using the inclusion criteria, the full text of 31 articles were obtained and details of those appearing to meet criteria were recorded in extraction tables. The first author and reviewer convened to compare the results of their respective searches. After excluding 19 of the 31 studies initially considered to meet criteria, 12 articles were finally agreed upon by the two assessors for inclusion (initial assessor consensus was 93%; where consensus was not reached, the second author was consulted). Reasons for exclusion were: duplicate articles of the same study (n = 8), follow up period not long enough (n = 2), did not record depression using appropriate assessment techniques (n = 2), and did not analyse/present data on predictors of work outcomes (n = 7). Figure 1 displays the results of the search strategy, in alignment with PRISMA guidelines. Papers included in the review were those published in English between 1994 and July 2009. Each article for final inclusion in the review was subject to assessment using a quality assessment inventory (Additional file 1). Quality assessment ratings are displayed in Table 2, where each article was graded using these criteria. Seven of the 12 articles were considered high quality, four moderate quality and one low quality. Collectively, the most common features of the articles were: well defined inclusion criteria, measurement selection and baseline data collection point, and use of multivariate techniques for data analysis. The least common feature of the articles was the reporting of a representative sample (four articles reported recruiting samples with males only). While measurements used for data collection were clearly documented, in most instances a justification for selection was not given.

**Table 2:** Quality of articles assessed using a framework for assessing internal validity [26]

Author	High 12 or more	Moderate 10-11	Low 9 or less
Bhattacharyya (2007) [14]	✓		

Brink (2008) [30]	✓		

Fukuoka (2009) [28]	✓		

Engblom (1994) [27]	✓		

Ladwig (1994) [34]			✓

Mayou (2000) [9]		✓	

McGee (2006) [35]	✓		

Mittag (2001) [33]		✓	

Soderman (2003) [5]		✓	

Soejima (1999) [31]	✓		

Sykes (2000) [32]		✓	

Samkange-Zeeb (2006) [29]	✓		

### Population and design

Articles included a collective total of 2795 participants who were employed at the time of their cardiac event, of working age (18+ [retirement age differed between countries]), recruited from an acute hospital setting with one of the following diagnoses: MI, ACS or CAD (including those undergoing cardiac interventions: Coronary Artery Bypass Graft (CABG), Percutaneous Transluminal Coronary Angioplasty (PTCA)). Data were derived from prospective cohort or longitudinal studies using prognostic variables, with the exception of one randomised controlled trial of a cardiac rehabilitation intervention [27]. Timing of classification of participant baseline depression ranged from hospital admission, upon stabilising of condition, immediately prior to discharge, pre surgical intervention, beginning of rehabilitation program, three days post discharge, 7-10 days post discharge, 17-21 days post discharge and two months post discharge. It was not possible to determine the average length of time since infarct as a result of this variation. Follow up assessment points used in the studies ranged from six months, eight months and 12 to 13 months.

### Depression Measures

Studies recorded depression outcomes using validated instruments. The most commonly used instrument was the Beck Depression Inventory [5,14,27,28], followed by the Hospital Anxiety and Depression Scale (HADS) [9,29,30], Cornell Medical Index [31], Subscale of Minnesota Multiphasic Personality Inventory (MMPI) [32], Center for Epidemiologic Studies Depression Scale German version (CES-D-ADS) [33] and a validated 12 item depression measure [34]. One study used both HADS and BDI Fast Scale (BDI-FS) [35] to assess depression, but after independent analysis of the measures, reported that HADS was superior to the BDI-FS in predicting RTW (p = 0.026), the results of the former instrument were included in the review.

### Definition of Work

RTW data were collected via self report (participant interview or questionnaire) in all studies to determine work status post MI. One study also used work data from a Social Insurance Institution Registry [27] to validate participant self report. Although the data collection method was consistent between studies, there was wide variation regarding the definition of RTW and the subsequent questions asked to participants (Table 3). Broadly, work resumption was defined as either a reported date of RTW or a positive response to the question: "Have you returned to work?". Only two studies considered RTW to be defined by a tangible time frame (i.e. "hours per week", returned at 100% of hours pre infarct). In the absence of these data, it was not possible to calculate mean time between cardiac episode and RTW. In a further attempt to ascertain work status, over half of studies (n = 7) collected information on work hours (full or part time) and almost one quarter provided estimates of current and pre-infarction activity. Additional information collected included: intent to RTW, disability, profession, early retirement, sick leave, job strain and organizational characteristics. One study did not provide a sufficient definition of RTW in its methodology but expressed findings as proportions of participants "seeking" and "returning" to work at follow up [9].

**Table 3:** Summary of population, data collection, endpoints of studies included in review

Authors	Population	Assessment points	Depression measure	Definition of Return to Work (RTW)
Bhattacharyya (2007) [14]	N = 126 ACS patients	7-10 days after admission, 12 months	BDI	Patients were asked when they had started work again and whether they were working full time or part time.

Brink (2008) [30]	N = 88 MI patients	4-6 months	HADS	Questionnaire about gainful employment, unemployment, early retirement, sick leave before and after MI

Fukuoka (2009) [28]	N = 198 ACS patients	During hospitalisation, 2 and 6 months after hospital admission	BDI	Questionnaire about work status and the date participants returned to work. RTW was defined as starting back at work for more than 20 hours/week.

Engblom (1994) [27]	N = 102 CABS male patients	Before CABG, 2 and 8 months after	BDI	Questionnaire, interview about work status (defined as paid employment, full or part time) and check of registry of Social Insurance Institution

Ladwig (1994) [34]	N = 377 MI male patients	17-21 days after event, 6 months	Validated 12-item version of depression composed of three subscales with rank-ordered ratings from 1 to 3	Patients were asked to complete a questionnaire about vocational and social status at the time of participation. 'Have you returned to work?'

Mayou (2000) [9]	N = 344 MI patients	3 days after admission, 3 and 12 months	HADS	Insufficient

McGee (2006) [35]	N = 363 ACS	In hospital, 12 months	BDI -FS, HADS-D	Questionnaire about RTW (full or part time employment)

Mittag (2001) [33]	N = 119 males post MI or CABG patients	During hospitalisation, 12 months	CES-D/ADS Depression	Postal questionnaire, asking whether participants had resumed their occupations, if they were working in their former job or had changed to some other workplace, and if they were working full time or not.

Soderman (2003) [5]	N = 198 CABG, PCTA patients	"Start of program," end of four week residential stay, 12 months	BDI	RTW was measured in two different ways, (a) RTW at full-time (100% of earlier working hours), and (b) RTW at reduced working hours

Soejima (1999) [31]	N = 111 married males AMI patients	Average 24.8 days post admission (in hospital) Average 8 months	Cornell Medical Index, 6 item depression index	Three measures of RTW: whether participant had returned to work, interval in days between hospital discharge and resumption of work, and estimates of activity level at work compared with before MI

Sykes (2000) [32]	N = 149 MI Patients	Baseline was pre discharge upon stabilising of condition and again at 12 months	Subscale of MMPI	Employment status was defined as returned to work or not, with information collected on patient occupation, Social Economic Status and work strain

Samkange-Zeeb (2006) [29]	N = 620 CHD patients	Beginning of rehab, 6 and 12months post rehab	HADS (adjusted for Germany)	Current working situation and questionnaire on intention to RTW, disability and profession

### Impact of Depression on RTW

Depression was a significant predictor of failure or delay in RTW at 6-12 months in 7 of the 12 studies. These studies are outlined in Table 4 along with a summary of effect sizes, p values and confidence intervals regarding the likelihood of depressed patients returning to work after MI. Findings are expressed as estimated relative risk and adjusted odds ratios are presented. Potentially confounding variables controlled for in each regression model are detailed (commonly demographic, clinical and other variables previously found to influence RTW rates in these populations or those found to be significant as a result of univariate analysis).

**Table 4:** Summary of effect of depression predicting likelihood of RTW post-MI at 6-8 and 12-13 months

Author	Finding	Ratio	Depression severity	Estimate of relative risk	CI (95%)	P value	Variables included in multivariate analysis** (bold indicates significance)
**DEPRESSION SIGNIFICANTLY PREDICTED RTW**							

**6-8 MONTHS**							

Fukuoka (2009)[28]	As a time-dependent covariate, increases in depression score predicted slower RTW at 6 months	Adjusted Hazard ratio*	Moderate depression Severe depression	0.47 0.37	0.31-0.72 0.21-0.66	< 0.001 0.001	Age, sex, nationality, education, income, marital status, smoking, hyperlipidemia, Duke activity index score (physical functioning), job strain, **job satisfaction**, job security, **working hours ** **per week**, **shift work**, social support (from supervisor, co-workers)

Samkange-Zeeb (2006)[29]	Level of depression was significant predictor of RTW at 6 months	Adjusted Odds ratio	Borderline depression Clinical depression	0.62 0.28	0.35-1.12 0.14-0.58		**Age**, sex, profession, anxiety, **expectations ** **about work incapacity ** **and desire to RTW**

Soejima (1999)[31]	Depressed patients less likely to RTW at 8 months	Adjusted Odds ratio		0.15	0.02-0.87	< 0.031	**Age**, education, occupation, **personality ** **type **health locus of control

**12-13 MONTHS**							

McGee (2006)[35]	Baseline depression significantly predicted RTW at 12 months	Adjusted Odds ratio	HADS depression	0.2	0.06-0.6	0.007	Prior ACS, age and sex

Sykes (2000)[32]	Depression significant predictor of RTW at 12 months	Wald test		7.335 (df = 1)		0.0068	**Decision latitude**, **work social ** **interaction**, **age, medical prognosis** **(Coronary Prognostic Index)**

Samkange-Zeeb (2006)[29]	Level of depression was significant predictor of RTW at 12 months	Adjusted Odds ratio	Borderline depression Clinical depression	0.35 0.24	0.18-0.68 0.11-0.49		**Age**, sex, **profession**, anxiety, **expectations about ** **work incapacity ** **and desire to RTW**

Soderman (2003) [5]	Clinical depression (BDI >16) predicted RTW at 12 months	Adjusted Odds ratio	Clinical depression Mild depression Clinical depression Mild depression	9.43 (fulltime) 2.89 (fulltime) 5.44 (reduced hours) OR not shown	3.15-28.21 1.08-7.70 1.60-18.53	<0.001 0.0300 <0.0068 0.7848	Gender, **age, ** **education**, exercise capacity

Bhattacharyya (2007) [14]	Every increase in BDI index reduced likelihood of RTW at 12-13 months	Adjusted Odds ratio		0.90	0.82-0.99	0.032	Age, gender, risk of cardiac event, heart failure, antidepressant use, **Arrhythmia during ** **admission, recurrent ** **cardiac events**

**DEPRESSION DID NOT SIGNIFICANTLY PREDICT RTW**							

**6-12 MONTHS**			**Significant ** **predictors**				

Brink [30]	Somatic health better predictor of RTW than mental health at 6 months	Adjusted Odds ratio	Physical health component score Footsteps per day	1.08 1.18	1.02-1.14 1.01-1.38	0.011 0.033	**Physical health**, age, **footsteps ** **per day**

Ladwig (1994) [34]	Depression as a significant predictor of RTW at 6 months (OR: 0.39, Cl 0.18-0.88), was lost after adjustment for age, social class, rehabilitation, recurrent infarction, cardiac events, helplessness (OR: 0.54 CI 0.22-1.31)		-				

Mayou (2000) [9]	No significant differences in RTW between distressed and nondistressed at 12 months		-				

Engblom [27]	At 12 months, patients' expectations of work, duration of absence from work before CABS and physical capacity of patients after surgery are important determinants of RTW after CABS	Adjusted Odds ratio	Self assessed work capacity at six months (Good vs Poor) Functional Class (Canadian CVD class I vs II-III) Patient expectation about work (RTW vs retire) Absence from work before the CABS (3 months or less)	8.5 6.7 6.4 4.9	2.3-32.0 1.8-24.5 1.6-26.0 1.2-20.2	0.003 0.006 0.013 0.032	Type of rehabilitation, previous MI, **expectations ** **regarding work**, physical strain of work, **duration of the ** **preoperative absence ** **from work**, basic education, professional education, socioeconomic status, preoperative BDI score, final work load at exercise test, **functional ** **class, patients' perception ** **of working capacity at ** **6 months after the CABS**.

Mittag [33]	Three variables predicted RTW at 12 months in 85% of all cases: (1) age, (2) patients' feelings about disability (3) physicians' views on the extent to which vocationally disabled	Adjusted Odds ratio	Age Self perceived disability Physician's view of disability	1.22 3.02 1.61	1.10-1.34 2.48-3.57 1.16-2.07	<0.01 <0.001 <0.05	Results of exercise testing, optimistic coping style, family income, negative incentives for RTW, physicians' subjective prognosis as to re-employment, patients' wish to return to work, **age, ** **self perceived vocational ** **disability, physician's ** **perception of patient disability**.

Of the studies to find depression a significant predictor of RTW, Fukuoka et al (2009) [28] and Bhattacharyya et al (2007) [14] found that depression not only significantly predicts work resumption but that a dose response relationship exists between severity of depression and likelihood of RTW, six to twelve months after a cardiac event. In regards to the impact of past history of depression on RTW, these were the only two studies to record depression which occurred pre-infarct. These studies reported disparate results. Fukuoka et al (2009) [28] found a significant difference in those with depressive history who RTW, when compared with those without (p < 0.05), while Bhattacharyya et al (2007) [14] found that depression experienced six month pre-infarct was not related to RTW at 12 months.

In these seven studies, other significant predictors of work resumption included demographic factors (age, education), organizational factors (job strain, decision latitude, social network at work, profession), clinical factors (recurrent cardiac events, arrhythmia), and individual factors (personality type, expectations, health concerns). Besides depression, age was the only variable to feature as a significant predictor in more than one study (n = 4).

Of the studies which failed to find depression a significant predictor of RTW, somatic health (OR 1.08 (CI 1.02-1.14; p = 0.011) and footsteps per day (OR 1.18 (CI 1.01-1.38; p = 0.033) [30] were significant predictors at six months. At 12 months, age (OR 1.22 (CI 1.10-1.34), self assessed work capacity at six months (OR 8.5 (CI 2.3-32.0; p = 0.003), physician's perception of disability (OR 1.61 (CI 1.16-2.07) [33], functional class (OR 6.7 (CI 1.8-24.5), and absence from work ≤ 3months (OR 4.9 (CI 1.2-20.2) [27] were all predictors of RTW. The only common predictor was patient perceptions; of health (self perceived disability; OR 3.02 (CI 2.48-3.57)) [33] and work (OR 6.4 (CI 1.6-26) [27]. However, many of these associations yielded wide confidence intervals.

Mayou (2000) found no significant differences in RTW of participants according to HADS score at 12 months [9], therefore a regression analysis was not reported for depression and RTW. Of the studies which found depression to be an independent predictor of RTW, five were considered high quality, compared with two of the studies which failed to find an effect.

## Discussion

The aim of the paper was to review whether depression remains a predictor of poorer work outcomes following MI, by reviewing the literature from the past 15 years. Our findings suggest that depression recorded between admission and up to two months post discharge can significantly predict poorer RTW outcomes 6 to 12 months after a cardiac event. There is also some evidence to suggest that increases in severity of depression can reduce likelihood of RTW. Age and patient perceptions of their illness or work performance were also shown to significantly predict RTW in these populations.

Our first finding is consistent with earlier studies conducted in the 1980 s [11,12], which found depression to be a strong determinant of work outcomes. Hlatky et al (1986)[12] found depression to predict work disability outcomes (χ^2 ^= 20, p < 0.00001), and loss of employment in the year following CAD (p = 0.006). More specifically, MÆland and others (1987)[11] found that RTW rates were strongly related to level of depression reported by MI patients at hospitalization (χ2 = 20.74, p < 0.05, *G = *-0.49) and 6 week follow-up (χ2 = 11.30 p < 0.05), and that this relationship was linear. Although this result appears in alignment with our second finding, it should be noted that a combined depression and anxiety measure was used in the MÆland study. The confounding effects of measuring these conditions using a composite instrument need to be considered.

Interestingly, both studies also found that alongside depression, patient perception was an important determinant of work status after a cardiac event. This was a finding observed in the current review, and elsewhere (Petrie et al, 1996)[36]. This raises questions about the role of cognition as a mediating factor in the relationship between depression and work.

Overall, commonalities between past and present studies may suggest that while the management of cardiac patients has changed in recent years, the factors influencing recovery and RTW identified over 15 years ago remain relevant. Determining the extent to which depression can predict major QOL outcomes post MI is important due to its clinical applications to rehabilitation. Modern rehabilitation programs should not only ascertain participant intent to resume work, but assess and treat depression in order to facilitate recovery. In depressed populations, patients receiving depression treatment such as anti-depressants or psychotherapy are significantly more likely to maintain paid employment over a 12-month period than those who do not [37]. Workplace initiatives targeting depression could potentially improve retention rates for employees exhibiting depression after returning to work post MI. These findings are of further value as it has been argued that identifying depression as a predictor of RTW could "give insight into mechanisms underlying an association between depression and cardiac mortality and morbidity" [9].

The review methods that we report on have two significant shortcomings. First, several articles in the review included samples comprising participants either recruited from cardiac rehabilitation or who had received a surgical intervention, post infarct. While it is acknowledged that this reflects modern management of cardiac patients, this may have confounded the representativeness of these samples. Those experiencing co-morbid depression are often less likely to attend rehabilitation programs, and report higher withdrawal rates [38]. As a result, depression may have been underrepresented in these samples. The inclusion of samples using participants who underwent surgical procedures may also have confounded results. These patients may experience added complications in the post operative period which prevent work resumption, or conversely, these procedures may promote better work outcomes, a finding which has been reported previously [39]. A further issue related to sampling was the lack of representativeness of female participants (one third of the studies had all male participants). For example, after a cardiac event, men have been found to have a greater likelihood of returning to work in a full time capacity and are less likely to report depression than females [40]. The inclusion of samples with only male participants may have both overrepresented RTW rates, and underrepresented the presence of depression. Female representation in this area of study is important when considering the proportion of those in paid employment at the time of MI has increased for both genders in recent times. For example in 1985, studies showed 34% of males and 18% of females were employed at the time of MI [41] compared with 65% and 32% respectively in 1999 [42], which may reflect demographic changes of workforce participation, or a decrease in the average age of a cardiac event.

If we compare the studies that did and did not find an association between depression and RTW post-MI, while no clear methodological differences were observed, failure to control for gender may have been a potential issue. Of the seven studies reporting depression as a predictor of RTW, one included males only, compared with three of the studies not reporting significant results. In fact, of the studies which failed to show depression as a significant predictor of RTW post-MI, only one controlled for gender (Mayou [9]), which may have had an impact upon results.

Second, the wide variation between definitions of RTW and depression measures may have undermined comparability of the studies included in the review. It should be noted that the variance in depression assessment instruments used in these studies also meant inconsistencies in time frames over which participants were asked to report their depression symptoms (for example, the MMPI assesses depression over a 12 month preceding period, while HADS assesses depression over a four week period), which has implications on results. Although not the focus of the review, there is evidence to suggest that depression assessment tools vary in their sensitivity to detect depression as a predictor of RTW [39]. Future studies in this area should consider this. Despite these limitations, our findings suggest that the majority of articles included in this review remained of moderate to high quality. In order to overcome the methodological limitations observed, we recommend the development and use of a brief, validated work measurement to capture employment outcomes, in order to enhance comparability of studies and allow for appropriate analyses of work outcomes. While depression was found to be a significant factor influencing RTW at both 6 and 12 months post MI, further research is required to determine the long lasting effects of cardiac depression on job retention. As the studies included in the review did not report assessing clinical depression using diagnostic instruments but rather self-report inventories, it remains unclear whether treating depression would improve vocational outcomes. While there is evidence that treating depression symptoms can improve vocational outcomes in primary care attendees (e.g. Lo Sasso et al [43]), this is yet to be demonstrated in CVD populations.

Therefore, we recommend that future clinical trials evaluating the effectiveness of post MI depression treatment use RTW as an endpoint. Furthermore, only two of the studies included in this review examined the impact of pre-existing depression on RTW rates. With evidence suggesting that depression outcomes (persistent major depression, subthreshold depression, or remission) are strongly associated with the probability of maintaining paid employment in depressed populations [44], further research is required into how work outcomes may differ according to types of depression in cardiac populations. Distinguishing between transient depressive symptoms following a life threatening cardiac event, (which, in many cases are only captured by self-report inventories), and more stable clinical depression may be useful for anticipating longer term effects on functioning.

## List of abbreviations

MI: Myocardial Infarction; RTW: Return to Work; ARIC: Atherosclerosis Risk in Communities; PCI: Percutaneous Coronary Intervention; ACE: Angiotensin-converting enzyme; ENRICHD: Enhancing Recovery in Coronary Heart Disease Patients; ACS: Acute Coronary Syndrome; CAD: Coronary Artery Disease; CABG: Coronary Artery Bypass Graft; CABS: Coronary Artery Bypass Surgery; PTCA: Percutaneous Transluminal Coronary Angioplasty; BDI: Beck Depression Inventory; BDI-FS: Beck Depression Inventory Fast Scale; CES-D: Center for Epidemiologic Studies Depression Scale; CES-D/AC: Center for Epidemiologic Studies Depression Scale, German version; HADS: Hospital Anxiety and Depression Scale; CVD: Cardiovascular disease; CHD: Coronary Heart Disease; CAD: Coronary Artery Disease; OR: Odds ratio; HR: Hazard Ratio; MMPI: Minnesota Multiphasic Personality Inventory; QOL: Quality of Life

## Competing interests

The authors declare that they have no competing interests.

## Authors' contributions

AO conceptualised the paper, synthesised, analysed and interpreted data, and wrote the original version of the manuscript. KS assisted with the inclusion/exclusion criteria, coding, synthesis and analysis of data and critically revised drafts of the manuscript. BO critically revised drafts of the manuscript. All authors approved the final version of the manuscript.

## Supplementary Material

Additional file 1 **Quality criteria**. Click here for file
