# Supplementary material for: Depression as a predictor of work resumption following myocardial infarction (MI): a review of recent research evidence
Source: Health Qual Life Outcomes. 2010 Sep 6;8:95. doi: 10.1186/1477-7525-8-95 (PMC2944344; doi:10.1186/1477-7525-8-95)
Supplement: Additional file 1 — Quality criteria. [file 1477-7525-8-95-S1.DOC]

Additional File 1. Quality criteria

**SAMPLE**

1. Inclusion criteria defined
2. Time of baseline data collection clearly stated
3. Representative sampling
4. Clinical/Demographic/ other important characteristics described
5. Study setting and site clearly described
6. Measures/data collection applied at appropriate assessment points for the research

**PROGNOSTIC INDICATORS**

1. The variables are clearly defined
2. Measures used are justified
3. The multifactorial nature of RTW is recognised.
4. Study uses standardise, valid instruments to take measurements

**ANALYSIS**

1. Multivariate techniques used to adjust for confounding variables
2. Analysis avoided “overfitting the data”
3. Prospective validation in another cohort was performed

**PATIENT FOLLOW UP**

1. RTW outcome was defined (or measure of absence)
2. Duration of follow up greater or equal to 6 months
3. Complete data on at least 80% of sample (measured at baseline)
4. Outcome measures were blinded (not revealed to patients)
